# *In Vivo* Corrosion Behavior of Biodegradable Magnesium Alloy by MAF Treatment

**DOI:** 10.1155/2021/5530788

**Published:** 2021-05-03

**Authors:** Xinzhe Gao, Chun Yu Dai, Qi Jia, ChuanYao Zhai, HaoYu Shi, Yifan Yang, Bing Cheng Zhao, HongXin Cai, Eui-Seok Lee, Heng Bo Jiang

**Affiliations:** ^1^The Conversationalist Club, School of Stomatology, Shandong First Medical University & Shandong Academy of Medical Sciences, Tai'an, Shandong 271016, China; ^2^Department of Oral and Maxillofacial Surgery, Graduate School of Clinical Dentistry, Korea University, Seoul 02841, Republic of Korea

## Abstract

Coating treatment plays an irreplaceable role in propelling the clinical application of magnesium alloys. This experiment was designed in order to observe the anticorrosion behavior of magnesium fluoride coating in rats. The MgF_2_ layer was prepared on the surface of AZ31 magnesium alloy in saturated NH_4_HF_2_ solution by microarc fluorination (MAF) at 190 V. The cross-sectional SEM, EDS, and XRD analysis indicated that the alloy surface was covered with MgF_2_. Meanwhile, SEM observation was used to compare the magnesium alloy samples before and after treatment, and it was found that the samples after coating were flatter and smoother. Two sets of experiments were carried out with the subjects, 6-week-old male rats. So that the untreated AZ31 samples and the microarc fluorinated AZ31 samples could be buried under the muscle layer individually. The volume changes and surface morphology of the corroded samples were monitored dynamically using micro-CT over a 16-week period *in vivo*. Comparison of results between the two sets of samples presented that the corrosion of the microarc fluoridated samples was much slower than that of the untreated ones. The MAF coating was shown to be effective in controlling the corrosion rate and progression of the magnesium alloy.

## 1. Introduction

As a biomaterial, magnesium (Mg) and magnesium alloys are promising for medical applications. Magnesium and magnesium alloys provide with high specific strength and desirable biocompatibility as implantable materials. Moreover, the density of magnesium and magnesium alloys is similar to that of the human bone [1, 2]. Nowadays, clinical practice commonly uses materials that primarily provide long-term structural stability, such as cobalt chromium alloys, titanium alloys, stainless steel, and other metals. However, because these materials are alien to the body, allergic or inflammatory reactions during treatment are also very common in subsequent recovery [3]. The process of bone remodeling will also be severely disrupted if a stress shielding effect occurs. Even secondary fractures could occur after implant removal surgery [4, 5]. It is worth noting that secondary surgeries after fracture healing will undoubtedly increase pain and medical costs. Magnesium materials, meanwhile, may avoid the aforementioned side effect. Consequently, magnesium alloy seems to be a more desirable option. Nevertheless, one outstanding drawback of magnesium materials is the rapid corrosion rate in human body. It will lead to problem such as an increase in the osmotic pressure of the local microenvironment, which may decrease the whole mechanical strength [1]. Therefore, it is a standing concern for scholars to control the corrosion rate of magnesium alloy.

The composition modification and alloy surface treatment can be used to slow down the corrosion progress of magnesium alloys. The former can improve its corrosion resistance and mechanical properties but not its bioactivity [6]. Currently, the commonly used surface modification methods are ionic, carbon treatment, fluoride treatment, etc. At this stage of research, electrochemical corrosion analysis has demonstrated that the magnesium fluoride (MgF_2_) layer can increase the polarization resistance of magnesium alloys [7–9]. In addition, in the existing studies to prove that the preparation of MgF_2_ coating has a certain influence on the corrosion resistance [7, 10, 11]. In addition, the biocompatibility of the MgF_2_ coatings has been shown to be positive in cytotoxicity experiments. The negative effects of the magnesium alloy on the degradation and precipitation of hydrogen gas in body fluids have been mitigated to some extent [8, 12, 13]. A point of interest is that the antimicrobial action of the magnesium fluoride coating is another property that has been found in previous studies [3]. In corrosion experiments, the Mg^2+^ and F^−^ ions produced during the degradation of the alloy are not only nontoxic to the surrounding tissues but also have a nutritional effect on the formation of the bone [6]. Fluorine in bone tissue mediates the metabolism of calcium and phosphorus and helps build bone strength [14]. Mg alloys coated with MgF_2_ are highly resistant to corrosion *in vivo*. Additionally, its anticorrosive ability can may be controlled to meet the requirements for implant retention for an appropriate period of time. The sustainability of the MgF_2_ coating is evident.

Experiments have been carried out to prepare MgF_2_ coatings using the microarc fluorination (MAF) method [15–17]. A series of *in vitro* corrosion tests proved that the microarc fluoride treated magnesium alloy, compared to the untreated ones, has been significantly improved in anticorrosion properties. Moreover, the proliferation and adhesion rates of cells grown on the coating were advanced [15, 16]. This method is based on the principle of electrolysis. MgF_2_ layers are prepared on magnesium or magnesium alloy substrates using a certain voltage as a reaction condition in a fluorinated solution [17, 18]. The MAF method was followed in this experiment. The purpose of this work is to observe the corrosion control by the MAF coating under the rat-body-fluid environment. A 190 V volt-treated MAF coating was prepared, and its dynamic changes and degradation behavior were recorded inside the experimental subjects. Its pre- and postcorrosion morphology, composition, and corrosion resistance were evaluated. This study complements the experiment system for the preparation of microarc fluorinated magnesium fluoride coatings by *in vivo* experiments.

## 2. Materials and Methods

### 2.1. Materials and Pretreatment

In this study, the size of the AZ31 (Dongguan FeiTai Metal Products Co., Ltd., China) was approximately 70.0 mm [3]. Then, polish the casted samples to 1000 grids. The AZ31 chemical composition is indicated in Table 1.

The testing samples were treated with microarc fluorination (MAF) method. Fluorinated coatings were prepared on samples in saturated NH_4_HF_2_ solution using 190 V constant voltage DC mode. A closed circuit was formed with AZ31 as anode and graphite rod as cathode during electrification. Clean all the samples with distilled water three times and dried thoroughly. The samples were divided into two groups labeled “Bare” and “MAF.”

### 2.2. Coating Observation

The morphology and cross-sectional images of each surface of the sample were observed by scanning electron microscopy (specific model reference: FE-SEM, JSM-67000). Then, the basic composition was determined by the energy spectrometer (EDS). The Mg phases on the surface of MgF_2_ samples were measured at 40 kV and 30 mA by X-ray diffraction (XRD, Rigaku Ultima IV Japan), and the scan rate was 1/min.

### 2.3. Electrochemical Experiment

The Potential Dynamic Polarization (PDP) tests were performed with a constant potentiostat (VersaSTAT 3: 300) and commercial software (VersaStudio 2.44.). The electrochemical analysis cell included a classical three-electrode cell which contained the testing sample as a working electrode, a pure platinum rod electrode, and a reference electrode (Ag/AgCl/Sat-KCl+197 mV vs. standard hydrogen electrode). Here, 1000 mL of commercial Hank's Balanced Salt Solution (HBSS) solution (WELGENE Inc., Korea) was used as the electrolyte and placed in a double-walled beaker. Maintain the temperature of the electrolyte at 37 ± 1°C using a recirculating water heater. The scanning rate of PDP measurements was 3 mV/s.

### 2.4. Animal Experiments

#### 2.4.1. Experimental Design

*In vivo* experiments were conducted with respect for animal ethics and animal protection guidelines. Six 6-week-old male rats (New Zealand) with an average weight of 180 g were randomly selected for this study. The rats were divided into two groups; one group was experimented with the Bare, and the other group was experimented with the MAF. After 16 weeks, the rats were euthanized, and the samples were removed.

#### 2.4.2. Surgical Procedures

**Figure 1:**
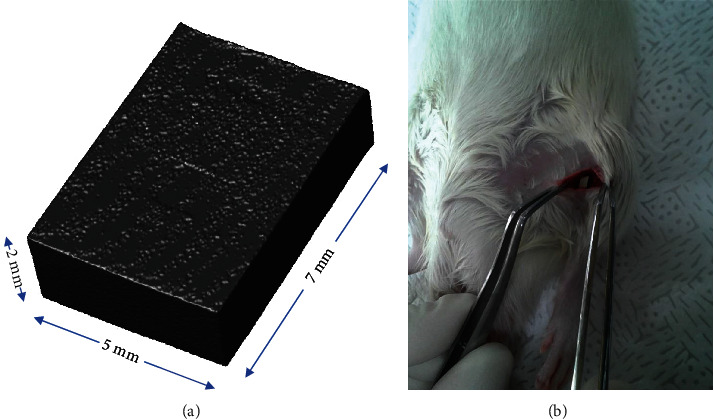
In vivo corrosion control samples and intraoperative diagrams in rats. (a) Stereoscopic imaging of the sample and annotation of the sampling size. (b) Preparation of an in vivo corrosion model in rats and surgical burial of the sample.

Firstly, the rats were anesthetized with isoflurane (Isolurane inhalation anesthetic solution; Pizer Japan Inc., Tokyo, Japan). Peel off the skin, fascia to expose the anterior tibial muscle. A sample was buried between the right tibial muscle and periosteum by dental round-bar and a nontaper tissue bar. Then, press the implant and flushed with saline, which can be seen in Figure 1. The wound was closed by suture needle (Nylon suture 5-0; Natsume seisakusho Co., Ltd.). After suturing the incision, put the rat on the pad until they recovered from the anesthetic state. For postoperative analgesia, 0.5 mL of transaminopurine hydrate (sulpyrine injection 250 mg NP; Nipro Pharma Corporation, Osaka, Japan) was added to in rats' drinking water as anti-inflammatory and analgesic for two weeks. At the end of the experiments, the rats were anesthetized with isoflurane (Pizer Japan Inc.) and euthanized by inhalation of CO_2_. The samples were removed from each rat.

### 2.5. Corrosion Analysis *In Vivo*

Samples from the fourth week were scanned by micro-CT (SkyScan1076, Bruker). The micro-CT scanner was set up with 100 kV voltage, 100 *μ*A current, 360-degree rotation, and 17.6 *μ*m pixel size. After the rats were anesthetized, the scan started 1 cm above the implant and ended 1 cm below it. After removal of the samples at week 16, the samples were scanned again. Both the images (2D and 3D) and the volume data were obtained by the program (CTAn-CTVol 1.10).

The volume loss percentages were calculated as:

(1) $$\begin{array}{c} \text{Volume loss}\%=\frac{V_{1}-V_{2}}{V_{1}}\times 100\%. \end{array}$$

*V*_1_ is the volume before implantation, and *V*_2_ is the volume after 16 weeks. In order to better visualize the *in vivo* morphology at week 16, the samples were washed in chromic acid (H_2_CrO_3_) and dried. The surface was observed by SEM.

## 3. Results

### 3.1. Specimen Surface Morphology

**Figure 2:**
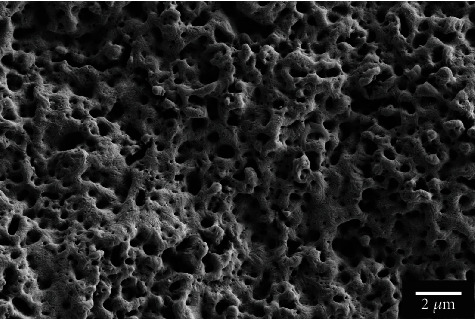
FE-SEM observing surface morphology after MAF coated.

Figure 2 illustrates the surface morphology of the MAF group under scanning electron microscopy before it was placed in the body. The surface of MgF_2_ coating was uneven and porous, coral-like.

**Figure 3:**
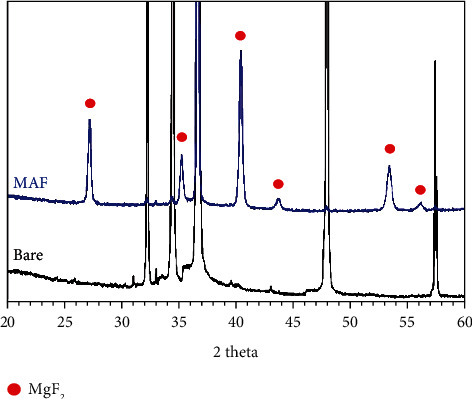
XRD patterns of the Bare and the MAF.

**Figure 4:**
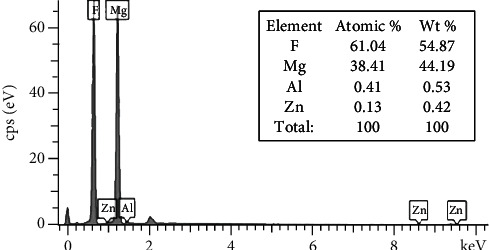
EDS spectra of the MAF coating and percentage of element composition.

Figure 3 presents the XRD curves of the Bare and the MAF sampling groups. Comparison of the untreated with the JCPDS standard card indicates that the coating of the treated sample consists of quadrilateral MgF_2_ (JCPDS No. 41-1443) mainly. EDS analysis showed that the coating surface was dominated by F and Mg elements, which accounted for 61.04% and 38.41%, respectively, as shown in Figure 4.

**Figure 5:**
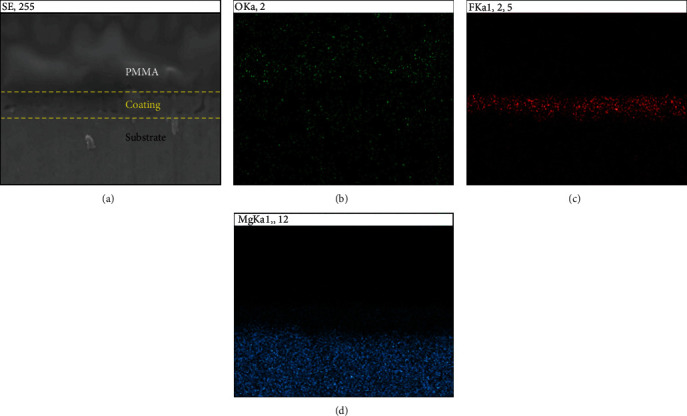
Cross-sectional electron microscopy images and energy diffraction spectra of the MAF coating. (a) Cross-sectional morphology observed under the SEM. Cross-sectional elemental mapping assay of the coating by EDS. (b–d) The results of the distribution of the three elements, oxygen, fluorine, and magnesium, respectively.

Figure 5 gives the SEM scan of the cross-section of the MAF group. From Figure 5(a), it can be seen that a layer appeared between the Mg alloy substrate and PMMA, i.e., on the surface of the base. According to the EDS mapping analysis, F elements were only distributed in the upper layer of the samples (Figure 5(c)), averaging 4.26 *μ*m thick. The middle part of the coating was more evenly spaced. The density of fluorine elements decreased as closer to the substrate, and the Mg elements in this depth were relatively sparsely distributed compared to the deeper part (Figure 5(d)). A combination of Figures 5(c) and 5(d) reveals a simultaneous distribution of Mg and F in this domain.

### 3.2. Corrosion Experiments

**Figure 6:**
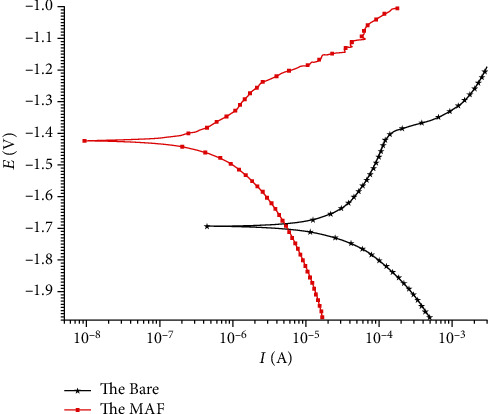
Potentiodynamic polarization curves of the Bare and the MAF samples.

Figure 6 is the empirical result of the dynamic potential polarization (PDP) experiment. Resistance properties can be reflected by the corrosion potential of the samples (*E*_corr_) and the current density (*I*_*d*_). The Bare group showed a corrosion current density of approximately 4.0 × 10^−5^ A. The *I*_*d*_ of the MAF group was about 2.1 × 10^−6^ A, which was about 20 times lower than the former. Significantly better corrosion resistance of the treated specimens can be interpreted.

**Figure 7:**
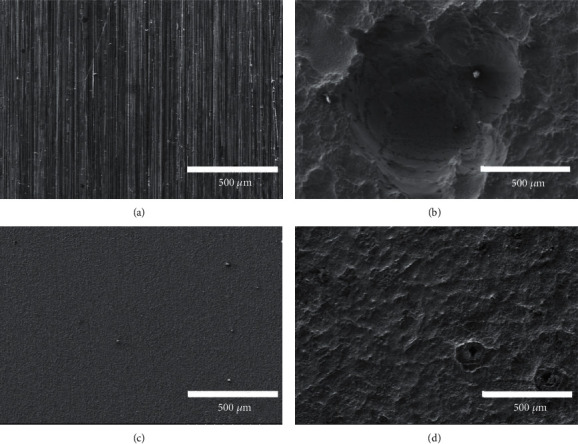
Observations of in vivo experimental samples before and after corrosion using FE-SEM. Surface morphology of (a) Bare, 0 week; (b) Bare, 16 weeks; (c) MAF, 0 week; and (d) MAF, 16 weeks.

The surface images obtained by SEM (Figure 7) reveal that the Bare before implantation (Figure 7(a)) was a line shape produced by grinding, whereas the MAF (Figure 7(c)) had a smoother surface view. After removal at week 16, significant pitting was observed on the Bare surface (Figure 7(b)). Scan results displayed a corrosion range of approximately 0.94 mm in diameter. The MAF surface only appeared to be water-washed soil with a few occasional pitting holes, which can be read from Figure 7(d). The surfaces of the Bare were more uneven due to more localized corrosion in comparison.

### 3.3. Micro-CT Scanning

**Figure 8:**
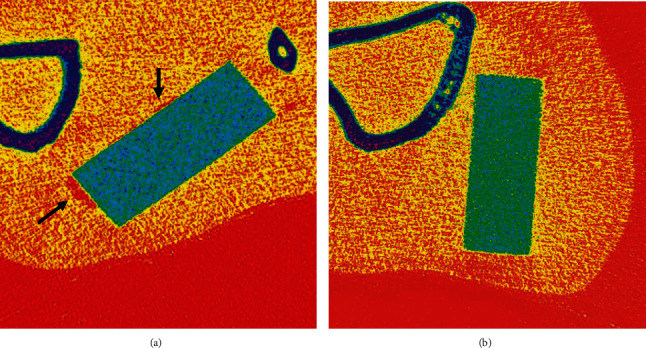
2D results of the micro-CT scan of the sample (a) Bare and (b) MAF burial site at week four.

Figure 8 depicts the imaging of two groups of samples *in vivo* at week 4 using micro-CT. The large gas cavity was clearly visible in the Bare group at the location indicated (Figure 8(a)), and there were more small gas cavities around the sample, so that the color spots were sparse. The MAF group, on the other hand, had denser imaging around the sample, proving that the gas production was pretty slow.

**Figure 9:**
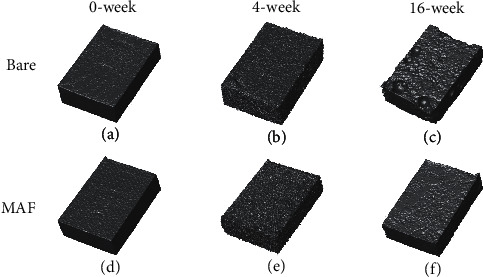
Micro-CT scans of samples from in vivo experimental procedures at selected time points, 3D images.

Figure 9 contains the results of micro-CT imaging of the specimens at weeks 0, 4, and 16, respectively. At week 4, a corrosion pit appeared in the lower left corner of the Bare samples and tended to enlarge. At 16 weeks, the sampling from the Bare group had other corrosion pits in addition to the one in the lower left corner, the largest of which was nearly 1 mm in diameter. In addition to this, it can be noted that the rectangular form becomes incomplete. Nevertheless, the MAF group had only a few pitting-like indentations on the surface of the sample. Judging from the results of week 4 and week 16, the MAF group would be less corrosive than the Bare group no matter what.

**Figure 10:**
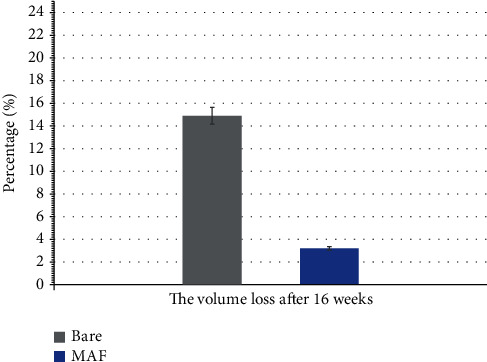
Volume change of in vivo experiments.

Figure 10 is a histogram produced from the volume loss after sample removal. The volume loss in the Bare group was 14.9% compared to 3.2% in the MAF group. The Bare group was approximately 4.67 times more likely to be in the MAF group.

## 4. Discussion

Mg alloy, as a biodegradable biomaterial, is commonly used to provide short-term support during tissue recovery, and its potential for medical applications is recognized [19–22]. Nevertheless, it has been demonstrated in existing studies that the rapid degradation rate and localized corrosion behavior of magnesium alloys hinder their clinical application [23, 24]. The existing problems may lead to a mismatch between the support period and the tissue recovery process or even fracture damage to the implant due to stress concentration [8, 25, 26]. In order to solve the fundamental problem, controlling the corrosion rate of magnesium alloys in the electrolyte *in vivo* has become a more widely studied objective.

Surface coating treatment of magnesium alloys has been used in many studies, and effective corrosion rate mitigation results have been obtained. The surface modification treatment can also improve the biocompatibility of magnesium alloys as biomaterials [8]. It is worth mentioning that the coating treatment used should satisfy the following points: (I) strong adhesion to magnesium alloy substrate, (II) provide corrosion resistance, and (III) no cytotoxicity. In addition, for stable degradation of the material within the physiological environment, the corrosion products should also have good biocompatibility assessment results [27]. Li et al. [28] found that Mg fluoride coating in 3.5% NaCl solution improved the bioactivity of the samples. Chiu et al. [29] conducted experiments using HBSS solution and found that MgF_2_ coating improved the corrosion resistance of the samples.

The corrosion tests in the *in vivo* environment were performed in combination with existing in vitro experiments on the MAF coating treatment. The composition, surface morphology, and electrochemistry of MAF-coated magnesium alloys have been evaluated to some extent by *in vitro* experiments in previous studies, with more satisfactory results [15–17]. In this experiment, the processed AZ31 samples were processed at 190 V constant voltage mode. Figure 2 shows the rough coral-like surface morphology of the microarc fluoride treated magnesium alloy, similar to previous MAF coatings at high voltages [15, 16, 30]. Figure 5(a) shows that the fluoride coating with a thickness of 4.26 *μ*m is evenly distributed on top of the magnesium substrate. The distribution of oxygen elements throughout the section in Figure 5(b) is, we speculated, due to the internal magnesium exposure caused by the casting and grinding. Oxidation reactions with the air will form a thin oxide film. Because of the short reaction time, there is not much oxygen content. By the infiltrative distribution of fluorine elements in Figure 5(c), it can be assumed that the surface of the substrate is transformed into a fluorinated layer during the electrochemical process. As the fluoride layer grows inward and outward simultaneously, the geometry of the magnesium alloy slightly changes [5]. This also leads to a relative decrease in the distribution density of magnesium in the part of the coating in contact with the substrate. The chemical reaction leads to the combination of magnesium and fluorine atoms, which changes the original distribution of the elements. In addition, the corrosion current density and corrosion potential of the postcoated samples are analyzed by PDP experiments. A significant enhancement of the corrosion resistance can be observed.

Based on the above facts, it can be stated that the coating on the magnesium substrate is prepared completely and that the coating has a certain corrosion limitation. However, the corrosion resistance of MAF-treated coatings in the *in vivo* environment has not been studied previously [15–17]. In a study by Chan [31], the need for loss analysis of implantable materials, especially magnesium alloys, is raised. Fischerauer et al. [32] stated that the surface morphology can affect the corrosion rate to some extent. The use of micro-CT to monitor the corrosion characterization of samples in a body fluid environment is used to determine corrosion behavior and corrosion rate. This allows assessment of the effectiveness of the coating treatment. A certain degree of sample contact damage is avoided, ensuring the accuracy of the scan results.

Magnesium alloys degrade rapidly in a body fluid environment, producing gases, composed mainly of hydrogen [23]. The process is influenced by the complex dynamics of the liquid environment in the organism. It has been shown that the presence of chloride ions (Cl^−^) makes the surface of Mg alloys more likely to exhibit the rapid pitting corrosion. Because the body fluid contains a large amount of Cl^−^, it can be converted into magnesium chloride (MgCl_2_) by replacement reaction after contact with magnesium hydroxide formed on the surface of magnesium alloy [23, 33].

In summary, the chemical equation of the reaction is as follows:

(2) $$\begin{array}{c} \text{Mg}\left(\text{s}\right)+2{\text{H}}_{2}\text{O}\to \text{Mg}{\left(\text{OH}\right)}_{2}+{\text{H}}_{2}\left(\text{g}\right)\\ \text{Mg}\left(\text{s}\right)+2\text{C}{\text{l}}^{-}\left(\text{aq}\right)\to {\text{MgCl}}_{2}\\ \text{Mg}{\left(\text{OH}\right)}_{2}\left(\text{s}\right)+2\text{C}{\text{l}}^{-}\to {\text{MgCl}}_{2} \end{array}$$

Obviously, there is a dynamic exchange of fluids between tissues, and their flow accelerates the corrosion process. It is worth mentioning that the damage of hydrogen bubbles to implants, such as causing a loss of mechanical integrity, has also been reported recently [34]. From the micro-CT imaging results (Figure 8(a)), it is evident that gas density belts appear around the untreated specimen. The presence of air cavities on the sample contact surface with body fluids represents the occurrence and product accumulation of the anodic dissolution process. This partly reflects the inappropriate degradation rate of magnesium alloys and the difficulty of wound healing due to bubbles. Although bubbles can adversely affect the implant, no further damage to the bone and surrounding tissues was observed, the same as in the subcutaneous magnesium implantation experiments of Kuhlmann et al. [35]. It has been shown that the hydrogen produced during the corrosion of magnesium-based materials can be removed and excreted to some extent [32, 36]. In a sense, the negative effects of corrosion behavior and bubbles can be minimized if the corrosion rate is kept within a safe range [25].

Fluorinated coatings have exhibited desirable properties in many previous studies. In addition to the most common aspects, its antimicrobial properties have been a noteworthy part of the research process since 1940 [37, 38]. Materials with bacterial resistance are highly desirable in medical implantation procedures [8, 39]. Scanning electron microscopy observation of the MAF coating in this experiment reveals similarities between the fluorinated coating and the previous study [15–17]. Therefore, we compared the results of the *in vivo* corrosion with the Hank's Balanced Salt Solution (HBSS) immersion experiments from previous studies. Similarities in the corrosion traces could be found [15–17]. Observation of Figure 9 SEM scan reveals an obvious corrosion pit, indicating localized corrosion of the uncoated magnesium alloy in a body fluid environment. This implies that corrosion does not develop uniformly at the interface between the alloy and the body fluid. Conversely, site-directed degradation corrosion has occurred at certain locations, and the corrosion process at that location is more advanced than at other planes at the same depth [40, 41]. When the rate of corrosion is greater than the carry-over excretion of body fluids, the gas accumulates and creates cavities. In other words, there is a high probability of gas localization at the location of the corrosion pits. This can also be demonstrated in conjunction with Figures 7(b) and 8(a). It can be inferred from the above facts that the presence of the MgF_2_ coating not only reduces the contact area between the magnesium alloy and body fluids but also effectively inhibits the rate of H_2_ production and reduces the damage of the implant material itself.

## 5. Conclusion

In this experiment, magnesium fluoride coatings were prepared on the surface of AZ31 magnesium alloy at 190 V constant voltages and evaluated for *in vivo* corrosion experiments, the following conclusions can be drawn.

1. Homogeneous and dense magnesium fluoride coatings are prepared and bonded to the magnesium alloy substrate
2. *In vitro* corrosion tests showed that the coating could significantly improve the corrosion resistance compared to bare magnesium alloy
3. *In vivo* experiments in rats showed that the coating provided protection to the magnesium alloy and significantly reduced gas generation and accumulation during the corrosion process. 190 V MAF coating exhibits good corrosion resistance in an *in vivo* environment

The combined results demonstrate that the MgF_2_ coating is effective in controlling the corrosion rate and progression of magnesium alloys, which shows its potential for medical implant material.

## Figures and Tables

**Table 1:** Chemical composition of AZ31 (mass fraction, %).

Al	Zn	Mn	Si	Mg
2.87	0.85	0.38	0.1	Balance

## Data Availability

The data used to support findings of this study are included within the article.
